# Prevalence of Common Mental Disorders Among Antenatal Women in Jaipur, India: A Comparative Study of Rural and Urban Populations

**DOI:** 10.7759/cureus.98856

**Published:** 2025-12-09

**Authors:** Surabhi Chakraborty, Anamika Tomar, Shiv Prakash Sharma, Vaseem N Baig

**Affiliations:** 1 Community Medicine, Jaipur National University Institute for Medical Sciences and Research Centre, Jaipur, IND; 2 Community Medicine, Rajasthan University of Health Sciences (RUHS) College of Medical Sciences, Jaipur, IND

**Keywords:** antenatal women, common mental disorders, maternal mental health, pregnancy, rural-urban comparison

## Abstract

Background

Common mental disorders (CMDs) among pregnant women represent a significant public health concern, particularly in low- and middle-income countries. Limited comparative data exist on the prevalence of CMDs between rural and urban pregnant populations in Rajasthan, India, which was the focus of this study.

Methods

A community-based, cross-sectional, comparative study was conducted from May to December 2022 in Jaipur. A total of 400 pregnant women (200 each from rural and urban areas) were recruited through simple random sampling from the field practice areas of the Urban Health Training Centre (UHTC), Jhalana Doongri, and the Rural Health Training Centre (RHTC), Dadiya village. Data collection utilised a structured socio-demographic questionnaire and the validated WHO SRQ-20 for CMD screening, with a cut-off score of ≥8 for CMD-positive.

Results

The overall prevalence of CMDs was 84 (21.0%) (95% CI: 17.08-24.92). Rural participants demonstrated a higher CMD prevalence at 48 (24.0%) (95% CI: 18.12-29.88) compared to urban participants at 36 (18.0%) (95% CI: 12.71-23.29), with an odds ratio of 1.44. However, this difference was not statistically significant (p = 0.177). Significant demographic differences were observed between populations, including caste distribution (p < 0.001), educational attainment (p = 0.001), family structure (p < 0.001), socioeconomic status (p < 0.001), and reproductive history (p < 0.001).

Conclusion

Both rural and urban pregnant women in Jaipur district experience a substantial CMD burden, necessitating the integration of mental health screening into routine antenatal care. Socio-demographic disparities between populations necessitate context-specific interventions tailored to local risk factors and cultural contexts.

## Introduction

Maternal mental health has emerged as a critical public health concern, especially in low- and middle-income countries (LMICs), where women face various biological, psychological, and social stressors during pregnancy [1]. Common mental disorders (CMDs), including depression, anxiety, and somatoform disorders, significantly contribute to a lowered quality of life during the antenatal period [2].

Global estimates indicate that 15% to 25% of pregnant women encounter CMDs, more so in LMICs [3]. In India, the prevalence is significant, with rates ranging from 9% to over 30%, differing by geographical location and methodology. Antenatal mental health is not prioritised in healthcare, resulting in CMDs routinely remaining undiagnosed [4].

Women experiencing CMDs during pregnancy are more prone to insufficient antenatal care, suboptimal nutritional conditions, and an elevated risk of obstetric problems, leading to negative newborn outcomes, including preterm birth, low birth weight, and heightened infant morbidity and mortality [5]. Besides physical health repercussions, CMDs correlate with disrupted mother-infant bonding, inadequate nursing habits, and hindered development in children, highlighting their intergenerational effects [6].

In India, various socio-cultural and demographic factors affect maternal mental health, such as poverty, low education, gender disparity, intimate partner abuse, and heightened susceptibility to chronic mental disorders during pregnancy [7]. Urban women may encounter pressures such as congestion, financial instability, and a lack of extended family support [8]. In contrast, rural women often face inadequate access to healthcare, economic hardship, and restrictive cultural norms. Comparative data on the frequency of CMDs in pregnant women in rural and urban areas are limited, especially in Rajasthan, where socio-demographic diversity and healthcare inequities may profoundly affect results [9].

This study aims to fill the evidence vacuum by comparing the prevalence of CMDs among pregnant women in rural and urban areas of Jaipur, Rajasthan. The study aims to highlight the need for the incorporation of maternal mental health screening and support into antenatal care programs, thereby enhancing maternal and child health (MCH) outcomes in the region.

## Materials and methods

Study design and setting

This community-based, cross-sectional, comparative study was conducted from May to December 2022 in Jaipur district, Rajasthan. The study was conducted in the field practice areas of the Department of Community Medicine, Rajasthan University of Health Sciences (RUHS) College of Medical Sciences, Jaipur, India, at the Urban Health Training Centre (UHTC), Jhalana Doongri, serving a 32,790 urban population, and the Rural Health Training Centre (RHTC), Dadiya village, serving a 33,127 rural population.

Ethical considerations

The study received ethical approval from the Institutional Ethics Committee of RUHS College of Medical Sciences, Jaipur (approval no. EC/P-63/2021). Additional permissions were obtained from the Chief Medical Health Officer (CMHO) of Jaipur II and the respective Medical Officers-in-Charge (MOICs) before data collection. Written informed consent was obtained from all participants in their preferred language (English or Hindi).

Study population and sampling

The study universe comprised all antenatal women residing in the field practice areas of UHTC Jhalana Doongri and RHTC Dadiya during the study period. The sample size was calculated using the formula for comparing two proportions: $n = \frac{Z^2 \big[ P_1(1-P_1) + P_2(1-P_2) \big]}{(P_1 - P_2)^2}$. Based on the article by Naaz et al. (2021) [10] and using a 95% confidence interval (Z = 1.96), the calculated sample size was 181 per group. Allowing 10% for non-response, the final sample size was 200 participants from each area, totalling 400 participants. Simple random sampling was employed for participant recruitment.

Inclusion and exclusion criteria

The inclusion criteria were antenatal women of all trimesters, aged 18 years or older, residing in the specified field practice areas, and providing written informed consent. Women were excluded if they had any obstetric complications in the current pregnancy (including hypertension, gestational diabetes, preeclampsia, preterm labour, advanced maternal age >35 years, teenage pregnancy, eating disorders, substance use, history of pregnancy loss, multiple pregnancies, anaemia, infections, malpresentations, placental disorders, low foetal weight, short stature, ectopic pregnancy, foetal disorders, and hyperemesis gravidarum), known psychological disorders, current use of medications such as antiepileptics, antidepressants, or central nervous system (CNS) stimulants, or critical or chronic illness.

Data collection tools

Data collection was performed using two structured instruments. Part A consisted of a pre-designed socio-demographic questionnaire collecting information on personal demographics (age, religion, caste, education), anthropometric measurements (height, weight, BMI), marital status and family characteristics, socioeconomic status using the Modified B.G. Prasad Classification (updated May 2021), obstetric history and current pregnancy details, and family support and living conditions. Part B utilised the Self-Reporting Questionnaire-20 (SRQ-20) by WHO, a screening tool for CMDs with an established sensitivity of 83% and specificity of 80% [11,12]. The validated Hindi version of the SRQ-20 has 20 yes/no questions, with scoring as 1 point for "Yes" and 0 for "No" (range: 0-20).

Operational definitions

For operational purposes, CMDs were defined as non-psychotic affective disorders classified in ICD-10 as “neurotic, stress-related, and somatoform disorders.” A CMD-positive was defined as an SRQ-20 score ≥8, and the antenatal period as a confirmed viable pregnancy of any gestational age before delivery.

Data collection and statistical analysis

Trained investigators conducted individual face-to-face interviews using structured questionnaires, with anthropometric measurements taken using standardised techniques. All data were collected in accordance with strict confidentiality protocols.

Data entry was performed in Microsoft Excel 2019 (Microsoft® Corp., Redmond, WA, USA) and analysed using IBM SPSS Statistics for Windows, Version 22.0 (Released 2013; IBM Corp., Armonk, NY, USA). Statistical methods included descriptive statistics (frequencies, percentages, and proportions for qualitative data; mean ± SD for quantitative data), as well as inferential statistics using the Chi-square test for categorical variables, with statistical significance set at a p-value ≤ 0.05. Prevalence calculations were performed with 95% confidence intervals, and odds ratios were used for risk assessment.

## Results

The overall prevalence of CMDs was 84 (21.0%) (95% CI: 17.08-24.92). Rural participants demonstrated a higher prevalence of CMDs at 48 (24.0%) (95% CI: 18.12-29.88) compared to urban participants at 36 (18.0%) (95% CI: 12.71-23.29), as shown in Table 1. Out of 200 rural participants, 48 (24.0%) women screened positive for CMDs, while 36 (18.0%) out of 200 urban participants were CMD positive. The odds ratio of 1.44 indicated that rural women were 1.44 times more likely to have CMDs compared to urban women. However, this difference was not statistically significant (p = 0.177, Chi-square = 1.823 with 1 degree of freedom) (Table 1).

**Table 1:** Prevalence of CMD by Location Odds Ratio = 1.44; Chi-square = 1.823 with 1 degree of freedom CMDs, Common Mental Disorders

Location	Total (N)	CMD Present	CMD Absent	Prevalence (%)	95% CI	p-value
Rural (Dadiya)	200	48 (24.0%)	152 (76.0%)	24.0	18.12-29.88	0.177
Urban (Jhalana Doongri)	200	36 (18.0%)	164 (82.0%)	18.0	12.71-23.29	
Total	400	84 (21.0%)	316 (79.0%)	21.0	17.08-24.92	

There were significant demographic differences between rural and urban populations. Age distribution was similar between groups (p = 0.566), with the majority, 226 (56.5%), in the 21-25 years age group. The religious distribution showed minimal variation, with 398 (99.5%) of participants identifying as Hindu. Caste distribution showed significant differences (p < 0.001), with rural areas having a higher representation of OBC (Other Backward Classes), 75 vs 50 (37.5% vs 25.0%), while urban areas had higher SC (Scheduled Castes) representation, 68 vs 38 (34.0% vs 17.5%). Educational attainment differed significantly (p = 0.001), with rural participants having lower educational levels, 80 (40.0%) having only primary education, compared to 43 (21.5%) in urban areas. Conversely, urban participants had higher graduation rates, 29 vs 15 (14.5% vs 7.5%) (Table 2).

**Table 2:** Socio-Demographic Characteristics of Study Participants by Location

Characteristics		Rural (n = 200)	Urban (n = 200)	Total (n = 400)	p-value
Age Group (years)	18-20	11 (5.5%)	9 (4.5%)	20 (5.0%)	0.566
	21-25	115 (57.5%)	111 (55.5%)	226 (56.5%)	
	26-30	70 (35.0%)	70 (35.0%)	140 (35.0%)	
	>30	4 (2.0%)	10 (5.0%)	14 (3.5%)	
Religion	Hindu	199 (99.5%)	199 (99.5%)	398 (99.5%)	0.368
	Muslim	0 (0.0%)	1 (0.5%)	1 (0.25%)	
	Others	1 (0.5%)	0 (0.0%)	1 (0.25%)	
Caste	General	44 (22.0%)	51 (25.5%)	95 (23.75%)	<0.001
	Scheduled Castes (SC)	35 (17.5%)	68 (34.0%)	103 (25.75%)	
	Scheduled Tribes (ST)	35 (17.5%)	28 (14.0%)	63 (15.75%)	
	Other Backward Classes (OBC)	75 (37.5%)	50 (25.0%)	125 (31.25%)	
	Others	11 (5.5%)	3 (1.5%)	14 (3.5%)	
Education	Primary	80 (40.0%)	43 (21.5%)	123 (30.75%)	0.001
	Middle School	49 (24.5%)	64 (32.0%)	113 (28.25%)	
	Higher Secondary	45 (22.5%)	53 (26.5%)	98 (24.5%)	
	Graduate	15 (7.5%)	29 (14.5%)	44 (11.0%)	
	Postgraduate	11 (5.5%)	11 (5.5%)	22 (5.5%)	

Gestational age distribution varied significantly (p < 0.001), with rural participants more commonly presenting in the third trimester, 83 vs 48 (41.5% vs 24.0%), while urban participants were more often in the first trimester, 61 vs 41 (30.5% vs 20.5%). Parity showed no significant difference (p = 0.080), though rural areas had slightly more multiparous women. Family structure differed significantly (p < 0.001), with rural participants predominantly living in joint families, 175 vs 150 (87.0% vs 75.0%), while urban participants had higher rates of nuclear family living, 43 vs 17 (21.5% vs 8.5%). The desire for a male child showed significant variation (p = 0.010), with rural women expressing a stronger personal desire for male children, 33 vs 19 (16.5% vs 9.5%). In comparison, urban areas showed higher in-law pressure for male children, 33 vs 19 (16.5% vs 8.5%) (Table 3).

**Table 3:** Obstetric and Family Characteristics by Location

Characteristics		Rural (n = 200)	Urban (n = 200)	Total (n = 400)	p-value
Present Gestational Age	First trimester	41 (20.5%)	61 (30.5%)	102 (25.5%)	<0.001
	Second trimester	76 (38.0%)	91 (45.5%)	167 (41.75%)	
	Third trimester	83 (41.5%)	48 (24.0%)	131 (32.75%)	
Previous Live Birth (Parity)	None	93 (46.5%)	98 (49.0%)	191 (47.75%)	0.080
	1	78 (39.0%)	87 (43.5%)	165 (41.25%)	
	2	20 (10.0%)	14 (7.0%)	34 (8.5%)	
	3	5 (2.5%)	1 (0.5%)	6 (1.5%)	
	>3	4 (2.0%)	0 (0.0%)	4 (1.0%)	
Type of Family	Living alone	8 (4.0%)	2 (1.0%)	10 (2.5%)	<0.001
	Nuclear family	17 (8.5%)	43 (21.5%)	60 (15.0%)	
	Joint family	174 (87.0%)	150 (75.0%)	324 (81.0%)	
	Three generation	1 (0.5%)	5 (2.5%)	6 (1.5%)	
Desire for Male Child	She desires	33 (16.5%)	19 (9.5%)	52 (13.0%)	0.010
	Husband desires	2 (1.0%)	7 (3.5%)	9 (2.25%)	
	In-laws desire	17 (8.5%)	33 (16.5%)	50 (12.5%)	
	No such desire	148 (74.0%)	141 (70.5%)	289 (72.25%)	

Socioeconomic status differed significantly (p < 0.001), with urban areas having a higher representation in Class I, 25 vs 5 (12.5% vs 2.5%), and rural areas having a higher proportion of participants in Class IV, 49 vs 37 (24.5% vs 18.5%). The BMI distribution varied significantly (p = 0.002), with urban participants exhibiting higher rates of underweight, 50 vs 22 (25.0% vs 11.0%), while rural participants had a more normal BMI distribution, 168 vs 140 (84.0% vs 70.0%). Overcrowding patterns differed markedly (p < 0.001), with urban areas experiencing more severe overcrowding, specifically an excess of two persons, 26 vs 2 (13.0% vs 1.0%), and an excess of three persons, 15 vs 1 (7.5% vs 0.5%). Family support systems showed significant differences (p < 0.001), with rural areas exhibiting stronger in-law support, 175 vs 134 (87.5% vs 67.0%), and urban areas displaying more diverse support patterns, classified as "others," 35 vs 0 (17.5% vs 0.0%) (Table 4).

**Table 4:** Socioeconomic Status and Living Conditions by Location

Characteristics		Rural (n = 200)	Urban (n = 200)	Total (n = 400)	p-value
Socioeconomic Status	Class I	5 (2.5%)	25 (12.5%)	30 (7.5%)	<0.001
	Class II	76 (38.0%)	74 (37.0%)	150 (37.5%)	
	Class III	69 (34.5%)	55 (27.5%)	124 (31.0%)	
	Class IV	49 (24.5%)	37 (18.5%)	86 (21.5%)	
	Class V	1 (0.5%)	9 (4.5%)	10 (2.5%)	
BMI Category	Underweight	22 (11.0%)	50 (25.0%)	72 (18.0%)	0.002
	Normal	168 (84.0%)	140 (70.0%)	308 (77.0%)	
	Overweight	8 (4.0%)	10 (5.0%)	18 (4.5%)	
	Obese	2 (1.0%)	0 (0.0%)	2 (0.5%)	
Overcrowding	Not present	149 (74.5%)	128 (64.0%)	277 (69.25%)	<0.001
	Excess of 1 person	48 (24.0%)	27 (13.5%)	75 (18.75%)	
	Excess of 2 persons	2 (1.0%)	26 (13.0%)	28 (7.0%)	
	Excess of 3 persons	1 (0.5%)	15 (7.5%)	16 (4.0%)	
	Excess of >3 persons	0 (0.0%)	4 (2.0%)	4 (1.0%)	
Family Support	Not supportive	3 (1.5%)	8 (4.0%)	11 (2.75%)	<0.001
	Only husband supportive	18 (9.0%)	23 (11.5%)	41 (10.25%)	
	In-laws supportive	175 (87.5%)	134 (67.0%)	309 (77.25%)	
	Maternal family supportive	4 (2.0%)	0 (0.0%)	4 (1.0%)	
	Others	0 (0.0%)	35 (17.5%)	35 (8.75%)	

There were significant differences in reproductive history (p < 0.001). Urban participants reported higher rates of previous pregnancy loss, 34 vs 14 (17.0% vs 7.0%), primarily due to higher rates of abortion/MTP, 33 vs 7 (16.5% vs 3.5%), while rural participants had higher rates of infant death, 7 vs 1 (3.5% vs 0.5%). Among those with previous losses, urban participants predominantly experienced spontaneous abortion, 33 vs 7 (68.75% vs 14.50% of total losses). In contrast, rural participants had equal distribution between spontaneous abortion, disease in the child, and pregnancy complications. The duration since the last loss showed no significant difference (p = 0.176), though urban participants tended to have more recent losses within one to two years, 9 vs 1 (4.5% vs 0.5%) (Table 5).

**Table 5:** History of Previous Loss of Child by Location

Characteristics		Rural (n = 200)	Urban (n = 200)	Total (n = 400)	p-value
Previous Loss of Child	No	186 (93.0%)	166 (83.0%)	352 (88.0%)	<0.001
	Abortion (spontaneous/MTP)	7 (3.5%)	33 (16.5%)	40 (10.0%)	
	Death of infant (0-1 year)	7 (3.5%)	1 (0.5%)	8 (2.0%)	
Number of Previous Losses	1	11 (5.5%)	32 (16.0%)	43 (10.75%)	0.214
	2	2 (1.0%)	1 (0.5%)	3 (0.75%)	
	3	1 (0.5%)	0 (0.0%)	1 (0.25%)	
	4	0 (0.0%)	1 (0.5%)	1 (0.25%)	
Duration Since Last Loss	<6 months	3 (1.5%)	5 (2.5%)	8 (2.0%)	0.176
	6 months to 1 year	10 (5.0%)	14 (7.0%)	24 (6.0%)	
	1-2 years	1 (0.5%)	9 (4.5%)	10 (2.5%)	
	2-5 years	0 (0.0%)	5 (2.5%)	5 (1.25%)	
	>5 years	0 (0.0%)	1 (0.5%)	1 (0.25%)	
Reason for Loss	Spontaneous abortion	7 (14.50%)	33 (68.75%)	40 (83.33%)	0.001
	Disease in a child	3 (6.25%)	1 (2.08%)	4 (8.33%)	
	Pregnancy complications	3 (6.25%)	1 (2.08%)	4 (8.33%)	

## Discussion

The current study identified an overall prevalence of CMDs of 84 (21.0%) (95% CI: 17.08-24.92) among antenatal women in the Jaipur district, which closely corresponds with the systematic review by Kalra et al. (2021), reporting a pooled prevalence of 21.87% (95% CI: 17.46-26.29) across 27 studies in India [13]. These data align with research by Prabhu et al. (2022) in Manipal (21.98%) and Dahiya et al. (2020) in Delhi (21%), indicating a uniform prevalence of mental health issues among pregnant women across various locations in India [14,15].

The prevalence identified in our study aligns with the findings of the National Mental Health Survey 2015-2016, which found that one in seven Indians experienced mental disorders, with 20% of affected individuals being pregnant women and new mothers. Our findings, however, diverge from research indicating elevated prevalence rates, as reported by Naaz et al. (2021) in Aligarh, Uttar Pradesh (38.8%), Sheeba et al. (2019) in Bangalore (35.7%), and Satyanarayana et al. (2011) in Bengaluru (33.5%) [10,16,17]. This heterogeneity can be ascribed to disparities in screening instruments, cultural contexts, and regional socioeconomic considerations.

Our investigation revealed that rural participants had a greater prevalence of CMDs, 48 (24.0%), compared to urban participants, 36 (18.0%), with an odds ratio of 1.44; however, this difference did not reach statistical significance (p = 0.177). This finding aligns with Naaz et al. (2021), who indicated increased odds of maternal CMDs in rural regions and reduced odds in urban mothers (OR = 0.63; 0.42-0.95) [10]. The elevated frequency in rural regions corresponds with global trends observed in LMICs, where rural inhabitants often face limited access to healthcare, financial constraints, and traditional cultural influences.

The rural prevalence of 48 (24.0%) in our study closely aligns with the findings of Raghavan et al. (2021) in rural Bihar (23.9%), indicating analogous burden patterns throughout rural India [18]. In contrast, research conducted in certain rural regions has indicated lower prevalence rates, as seen in studies by Jha et al. (2021) in rural Haryana (15.3%) and Rathod et al. (2018) in rural Madhya Pradesh (8.8%), suggesting regional disparities that local socio-cultural dynamics and healthcare facilities may influence [4,19].

The urban prevalence of 36 (18.0%) in our study aligns with Sidhu et al. (2019) in Punjab (18.0%) and is comparable to Sabita et al. (2019) in Puducherry (19.5%). However, it is lower than certain metropolitan studies, such as those by Johnson et al. (2018) in Bengaluru, which reported an incidence of approximately 5.8% [20-22]. Their research concentrated exclusively on third-trimester women, potentially elucidating the variance.

The pronounced disparities in caste distribution between rural and urban regions (p < 0.001) indicated crucial social stratification processes. Rural regions had a greater proportion of OBC residents, 75 vs 50 (37.5% compared to 25.0%), while urban regions showed a higher representation of SC populations, 68 vs 35 (34.0% versus 17.5%). This demographic heterogeneity may lead to disparate mental health outcomes, as socioeconomic deprivation and prejudice can heighten susceptibility to mental diseases.

Educational differences were evident, as rural participants had markedly lower educational attainment (p = 0.001). The observation that 80 (40.0%) of rural individuals possessed only primary schooling, in contrast to 43 (21.5%) in urban regions, corroborates the findings of Biaggi et al. (2016), who recognised poor educational attainment as a substantial risk factor for prenatal depression [23]. The educational disparity may contribute to the elevated prevalence of CMD in rural regions, as education serves as a protective factor by enhancing health awareness and coping strategies.

The prevalence of mixed family structures in rural regions, 174 vs 150 (87.0% vs 75.0%, p < 0.001), contrasts with global trends that favour nuclear families. This conventional family structure in rural areas was correlated with greater in-law support (87.5% versus 67.0%), potentially acting as a protective factor. This finding contradicts much research that has identified joint family living as a risk factor for CMDs, indicating that the quality of familial interactions may be more significant than the family structure itself.

The disparity in preference for male offspring (p = 0.010) between rural and urban regions suggests enduring gender biases, with rural women demonstrating a stronger personal preference, 33 vs 19 (16.5% vs 9.5%). In comparison, urban locales exhibited more in-law pressure, 16.5% vs 8.5%. This is consistent with Insan et al. (2022), who identified male gender preference as a significant predictor of prenatal depression (AOR 3.06, 95% CI 1.40-6.72) [24].

The pronounced socioeconomic discrepancies (p < 0.001) between rural and urban regions, with urban areas exhibiting greater representation in higher socioeconomic groups, illustrate overarching trends in economic development. The increased incidence of overcrowding in urban regions, paradoxically associated with reduced rates of CMDs, indicates that factors beyond physical living conditions affect mental health outcomes. This contrasts with the findings of Khan and Flora (2017) in Bangladesh, which identified household overcrowding as a significant risk factor for maternal CMDs [25].

The disparities in BMI distribution (p = 0.002), with urban individuals exhibiting higher rates of underweight (25.0% vs 11.0%), may indicate nutritional vulnerabilities in urban slum communities, despite improved healthcare access. This discovery necessitates further inquiry, as it challenges prevailing notions regarding the nutritional benefits of urban environments.

The elevated rates of prior pregnancy loss in metropolitan regions, 34 vs 14 (17.0% vs 7.0%) (p < 0.001), constitute an unforeseen observation. Urban participants indicated elevated rates of abortion/MTP (16.5% compared to 3.5%), whereas rural participants experienced greater infant mortality, 7 vs 1 (3.5% vs 0.5%). This pattern may indicate improved access to safe abortion services in metropolitan regions, while simultaneously implying distinct reproductive health issues. The prevalence of spontaneous abortion in urban regions (68.75% compared to 14.50% of total losses) corresponds with research by George et al. (2016), which found a history of miscarriage as a risk factor for prenatal depression [26].

The disparity in gestational age at recruitment (p < 0.001), with rural individuals predominantly presenting in later trimesters, 83 vs 48 (41.5% in the third trimester compared to 24.0% in urban areas), may indicate a lag in healthcare-seeking behaviour in rural regions. This pattern may affect CMD diagnosis rates, since Maheshwari and Divakar (2016) and Rehman et al. (2017) observed that the prevalence of depression escalates with the progression of pregnancy [27,28].

The variation in CMD prevalence between rural and urban regions (p = 0.177) may be ascribed to many reasons. The cross-sectional design limits our ability to determine temporal correlations, and reliance on a single screening instrument may fail to encompass the entire range of mental health issues. Cultural influences may also affect symptom expression and reporting, especially in rural regions, where mental health stigma is often more significant.

The significant variation in CMD prevalence among Indian research, as shown by Kalra et al. (2021) in their meta-analysis (I² = 97.53%), emphasises the relevance of regional and cultural influences on mental health expression [13]. This diversity necessitates state-specific, contextually appropriate interventions instead of universal solutions.

The significant prevalence of CMDs in rural, 48 (24.0%), and urban, 36 (18.0%), populations underscores the critical necessity of incorporating mental health screening into standard prenatal treatment. The results support the recommendations of the National Mental Health Policy 2014 and the Mental Healthcare Act 2017 for ensuring equitable access to mental health treatments. This study highlights socio-demographic gaps, underscoring the necessity for focused interventions that address socioeconomic determinants of mental health, such as education, economic empowerment, and social support systems.

The elevated CMD burden in rural regions, while not statistically significant, indicates the necessity for improved mental health interventions within rural healthcare systems. The socio-demographic disparities between rural and urban populations necessitate that prevention and intervention measures be customised to local contexts, targeting distinct risk factors inherent to each environment.

Study limitations

The study's limitations included a cross-sectional design, which limited causal inference; potential overlap of physical symptoms in the third trimester with psychological morbidities; limited generalisability due to the specific geographical area and study duration; and self-reporting bias inherent in questionnaire-based assessments.

## Conclusions

The distribution of castes, socioeconomic level, family structure, education, and reproductive history all showed differences between rural and urban populations. These findings underscore the necessity for context-specific mental health therapies, customised to local demographic attributes rather than standardised methods.

The significant prevalence of CMDs in both contexts requires the incorporation of mental health screening and support into standard prenatal care. This study presents the inaugural systematic evaluation of prenatal mental health in Rajasthan, providing foundational data for evidence-based policy formulation, with integration of mental health into currently existing MCH services through intersectoral coordination within existing health frameworks.
